# Enzyme Replacement Therapy for FABRY Disease: Possible Strategies to Improve Its Efficacy

**DOI:** 10.3390/ijms24054548

**Published:** 2023-02-25

**Authors:** Ilaria Iacobucci, Bruno Hay Mele, Flora Cozzolino, Vittoria Monaco, Chiara Cimmaruta, Maria Monti, Giuseppina Andreotti, Maria Monticelli

**Affiliations:** 1 Department of Chemical Sciences, Università degli Studi di Napoli “Federico II”, Complesso Universitario Monte Sant’Angelo, Via Cinthia, 80126 Napoli, Italy; 2 CEINGE Biotecnologie Avanzate “Franco Salvatore”, Via G. Salvatore 486, 80131 Napoli, Italy; 3 Department of Biology, University of Napoli “Federico II”, Complesso Universitario Monte Sant’Angelo, Via Cinthia, 80126 Napoli, Italy; 4 Institute of Biomolecular Chemistry ICB, National Research Council (CNR), Via Campi Flegrei 34, 80078 Pozzuoli, Italy; 5 Department of Environmental, Biological, and Pharmaceutical Sciences and Technologies (DiSTABiF), University of Campania “Luigi Vanvitelli”, Via Vivaldi, 43, 81100 Caserta, Italy

**Keywords:** Fabry disease, drug repositioning, interactome, GLA, Fabrazyme, Replagal, agalsidase α, agalsidase β

## Abstract

Enzyme replacement therapy is the only therapeutic option for Fabry patients with completely absent AGAL activity. However, the treatment has side effects, is costly, and requires conspicuous amounts of recombinant human protein (rh-AGAL). Thus, its optimization would benefit patients and welfare/health services (i.e., society at large). In this brief report, we describe preliminary results paving the way for two possible approaches: i. the combination of enzyme replacement therapy with pharmacological chaperones; and ii. the identification of AGAL interactors as possible therapeutic targets on which to act. We first showed that galactose, a low-affinity pharmacological chaperone, can prolong AGAL half-life in patient-derived cells treated with rh-AGAL. Then, we analyzed the interactomes of intracellular AGAL on patient-derived AGAL-defective fibroblasts treated with the two rh-AGALs approved for therapeutic purposes and compared the obtained interactomes to the one associated with endogenously produced AGAL (data available as PXD039168 on ProteomeXchange). Common interactors were aggregated and screened for sensitivity to known drugs. Such an interactor-drug list represents a starting point to deeply screen approved drugs and identify those that can affect (positively or negatively) enzyme replacement therapy.

## 1. Introduction

Mutations in the *GLA* gene cause the deficiency of lysosomal α-galactosidase activity and the consequent accumulation of its substrates (Gb3 and its derivative Lyso-Gb3), leading to Fabry disease (FD). FD is an X-linked lysosomal storage disorder and a multisystemic disease that strongly impacts the quality of life and reduces the life expectancy of the affected patients [1,2].

The organs affected the most in FD patients are the kidney (renal insufficiency), gastrointestinal tract (diarrhea and abdominal pain), central nervous system (stroke, transient ischemic attack, white matter lesion), heart (cardiac arrhythmia/failure, left ventricular hypertrophy), and the peripheral nervous system (neuropathic pain) [3].

FD shows a large phenotypic and genotypic spectrum without prevalent mutations [4]: more than 2000 different *GLA* mutations are known [5,6], most of which are family-specific. Among these, mutations causing a complete loss of enzymatic activity are associated with severe and early onset classical phenotypes. In contrast, those leading to a residual activity are associated with attenuated and late-onset phenotypes [7,8]. Females are not asymptomatic carriers, and cross-correction is not observed [9].

Two approved therapies are available for FD: enzyme replacement therapy (ERT) and pharmacological chaperone therapy (PCT). In addition, substrate reduction therapy, gene therapy, and nonsense suppression are pursued options, but they are still under investigation [10,11,12,13]. 

PCT for FD has been pioneered since galactose’s mechanism of action was described before introducing the term “pharmacological chaperone” [14]. In the early nineties, the beneficial effect of galactose treatment on AGAL mutant cell lines was identified [15,16]. Ishii and co-workers described the loss of stability by Q279E-AGAL, a mutant with similar kinetic properties to wt-AGAL. In vitro, such an unstable mutant was stabilized in the presence of galactose. Ishii et al. also found that intracellular AGAL activity increased upon high-concentration galactose treatment (100 mM) in COS-1 cells expressing the mutant gene and in patient-derived lymphoblasts. The hypothesis envisaged by Ishii and co-workers that galactose could represent a therapeutic strategy for FD was tested in vivo by Frustaci et al. in 2001. The authors described the treatment of an FD patient carrying a cardiac variant (G328R) with infusions of galactose, leading to a remarkable improvement in his clinical conditions. The author’s comments testify the importance of this improvement: “Cardiac transplantation was no longer required in this patient, because of the clinical improvement (from NYHA functional class IV to class I) during galactose-infusion therapy. The patient has returned to full-time work as a bus driver” [17]. A galactose analog was synthesized in 1999 by Fan and co-workers, stating that “1-deoxy-galactonojirimycin (DGJ), a potent competitive inhibitor of alpha-Gal A, effectively enhanced alpha-Gal A activity in Fabry lymphoblasts, when administered at concentrations lower than that usually required for intracellular inhibition of the enzyme” [18]. Since its first appearance, the scientific community’s attention has focused on its usage, and a large body of literature was produced until the FDA approval in 2018 [19]. DGJ, currently commercialized as Migalastat (Galafold^TM^) [20], is the only approved pharmacological chaperone for PCT. DGJ stabilizes AGAL, but it is also a strong inhibitor, so a discontinuous administration is recommended (“do not take Galafold on two consecutive days” is reported in Galafold’s instructions sheet). 

Although ERT has been applied since 2001, with a long clinical experience supporting its efficacy and safety, it is not free from critical issues: the therapy requires lifelong, frequent intravenous infusions and does not pass the blood–brain barrier. Furthermore, it may induce adverse infusion reactions, cause the formation of neutralizing antibodies that reduce treatment efficacy, and may have limited tissue penetration. Finally, ERT is expensive due to its dependence on a conspicuous amount (milligrams) of the recombinant enzyme (that is produced in engineered animal cells). Nevertheless, ERT is the only option for those patients who do not express *GLA* and for those genotypes that are not responsive to migalastat [21].

There are two forms of ERT available: agalsidase α, produced in human fibroblasts (Replagal^®^), and agalsidase β, produced in Chinese hamster ovary (CHO) cells (Fabrazyme^®^). A different dosage is recommended for the two: 0.2 mg/kg of agalsidase α every other week; and 1.0 mg/kg of agalsidase β every other week. agalsidase α and agalsidase β differ in their glycosylation, notably in the levels of the mannose-6-phosphate present, with the latter containing a higher percentage of the sugar [22].

In 2015, another recombinant protein was expressed in tobacco cells (pegunigalsidase α) [23] to circumvent some issues raised by agalsidase β and agalsidase α. Pegunigalsidase αis composed of two subunits of AGAL covalently bound by a chain of polyethylene glycol, which increases its stability and reduces its clearance, thereby extending its plasma half-life and allowing a monthly infusion [24,25]. This protein does not contain phosphorylated mannoses, yet it is captured by fibroblasts, suggesting an alternative uptake mechanism. It has been demonstrated that this rh-AGAL reduces substrate accumulation in animal models and has other positive effects [23]. However, it has not been approved yet.

Optimizing the approved ERT would be desirable since it would reduce either the amount of enzyme required or the frequency of the infusions. From this perspective, it could be interesting to search for ligands that stabilize rh-AGAL and identify druggable interactors of AGAL. The researchers who developed Migalastat (DGJ) were the first to introduce the idea of co-formulating ERT with a pharmacological chaperone [26]. However, DGJ is a strong competitive inhibitor (IC50 59 nM with endogenous AGAL [27]; Ki 39 nM [28]). Therefore, besides its stabilizing effect on AGAL, there would be activity inhibition as a side effect. As it is for DGJ, galactose both stabilizes and inhibits the enzyme, but it is a weak competitive inhibitor (Ki 16 mM, [28]) [16,29,30]. Thus, if co-administered with rh-AGAL, the inhibition would be minimized, and ERT would be effectively improved.

Herein, we provide a proof of concept that could pave the way for further studies on ERT improvement. We propose combining ERT with galactose, taking advantage of its properties as a pharmacological chaperone, or drugs acting on AGAL interactors.

## 2. Results and Discussion

### 2.1. ERT May Benefit from a Combination with Galactose Treatment

The high-molecular-weight (50 kDa) endogenous AGAL precursor is typically matured into the active form (46 kDa) before being transferred to the lysosome, and selectively released extracellularly [31,32]. The use of pharmacological chaperones relies on the possibility to improve this process, stabilizing the native enzyme and preventing its degradation. This is also true for the rh-AGAL, which are imported in a high-molecular-weight form, then processed in cells. As shown in Supplementary Figure S1, FD-derived immortalized fibroblasts (IF-NULL) were treated with agalsidase α or agalsidase β and incubated at increasing times. Immunoblot revealed a progressive increase in the 46 kDa band (lower molecular weight) over time. Additionally, the ratio between the 46 kDa and the 50 kDa bands (lower/higher) increased over time, confirming the maturation process.

**Figure 1:**
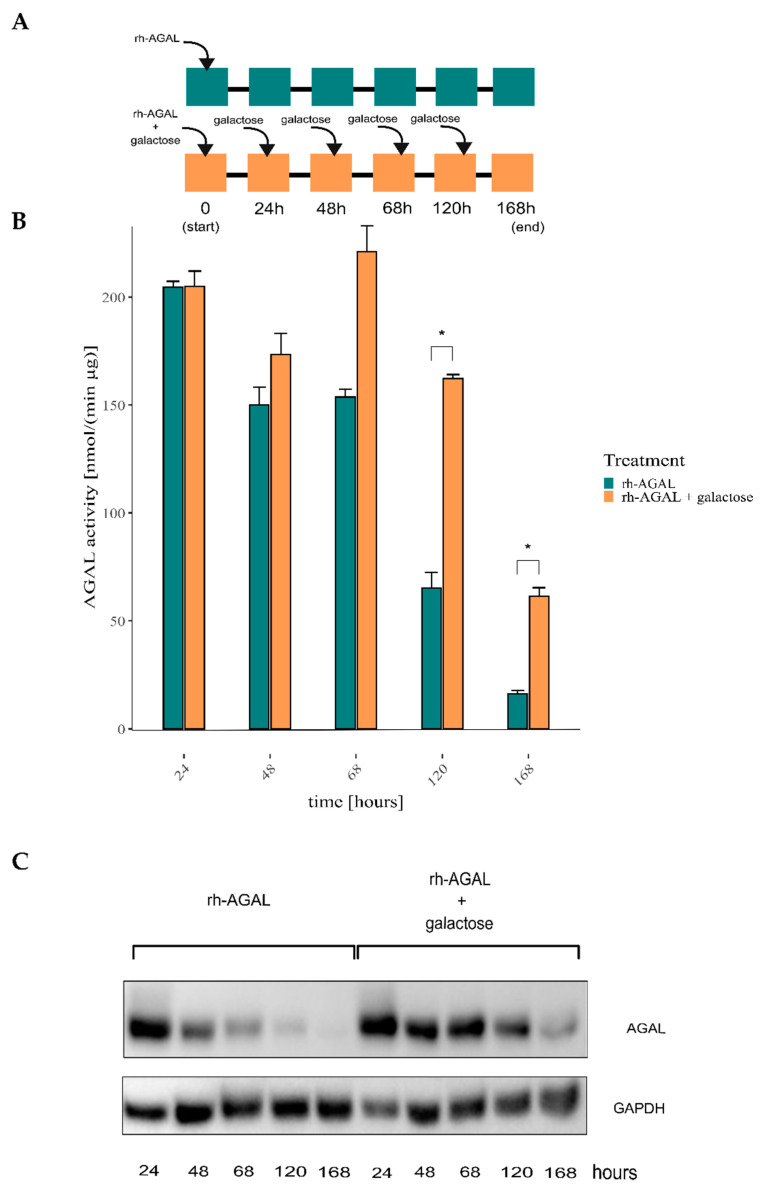
Galactose co-administration improves rh-AGAL stability in FD cells. Patient-derived fibroblasts defective of AGAL (IF-NULL) were treated with 1.5 µg/mL rh-AGAL (agalsidase β) in the presence or the absence of 100 mM galactose; galactose was then added in the culture medium for seven days, as sketched in panel **A**. Cells were collected and lysed, and AGAL quantity was assessed via enzyme activity assay (panel **B**) and immunoblot (panel **C**). The improvement of rh-AGAL stability over the seven days was detected (two-tailed unpaired *t*-test, n = 2; * = *p* < 5 × 10^−2^; panel **B**: 120 h, adj. *p.*0.0154; 168 h, adj. *p.*0.0233).

FD-derived immortalized fibroblasts (IF-NULL) were then treated with 1.5 μg/mL rh-AGAL (agalsidase β) in the presence or absence of galactose (serum concentration reached upon infusion: 2–3.5 μg/mL [33]). The treatment was conducted over seven days with galactose renewal, as depicted in the workflow (Figure 1A). Figure 1 shows that galactose does not influence the uptake of the rh-AGAL. However, it improves its intracellular stability over time, as assessed by enzymatic activity assay (Figure 1B) and immunoblot (Figure 1C). An accurate evaluation of the timing and dosage regimen in galactose administration would pave the way to combined therapy, i.e., ERT plus galactose.

In this frame, we incubated agalsidase α at 37° in fetal bovine serum (FBS) in the presence of galactose. Different concentrations were tested, up to 10 mM; the rationale behind this choice was the possibility of obtaining a stabilizing effect at lower dosages than the one tested in cells (100 mM), which had already shown its effect. Besides the inactivation observed in time, we were surprised to register an increase in enzymatic activity in the presence of low-concentration galactose (Supplementary Figure S2). This result is comparable with findings shown by Guce and co-workers, who measured the enzymatic inhibition of rh-GAL by both the pharmacological chaperones (DGJ and galactose) [28]. They showed a higher enzymatic activity in the presence of low-concentration galactose compared to the activity measured in its absence. This result suggests an activation driven by low-concentration galactose and underlines the need for further investigations in the use of galactose for FD.

The therapeutic use of galactose currently includes its use as a co-adjuvant for attenuated vaccines and treating constipation, hepatic encephalopathy, and hepatic coma [34]. Additionally, many clinical trials focusing on galactose as a drug or a dietary supplement have been reported for many different diseases, particularly congenital disorders of glycosylation [35,36]. Oral galactose is administered in high dosages, ranging from 0.5–1.5 g/kg/day [37]. A 2.57 ± 0.53 mM plasma concentration of galactose is described to be reached in one hour after the oral administration of 0.72 g/kg galactose [38]. The efficacy of the oral administration of DGJ in combination with ERT had previously been proved [39].

### 2.2. Identification of AGAL Interactors

Another way to improve ERT would be to act indirectly by targeting AGAL interactors. To this end, we focused on identifying and comparing proteins interacting with agalsidase β or agalsidase α during their internalization by AGAL-defective fibroblasts (IF).

Preliminary experiments were conducted to define the treatment conditions, as shown in Supplementary Figure S1 and discussed in the previous paragraph. IF cells were treated with 3 µg/mL rh-AGAL (agalsidase α or agalsidase β), and an uptake time-course was monitored over 6 h. The progressive increase in the internalized and matured rh-AGAL was observed.

**Figure 2:**
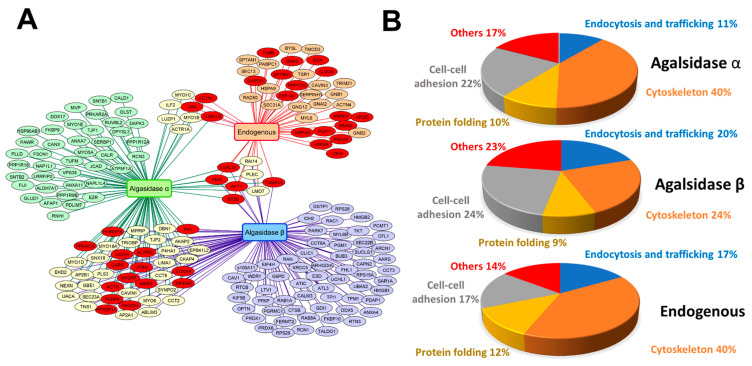
Graphical representation of AGAL and rh-AGAL interactomes and their functional clustering. Panel **A**: Cytoscape network representing putative AGAL interactors, identified by their MGI gene symbols. In the graph, nodes represent proteins, and lines represent interactions. The nodes are selectively identified as endogenous AGAL, agalsidase β and agalsidase α; protein partners are reported in light red, light violet, and light green, respectively. The shared interactors between different conditions are shown. Red ellipses mark druggable proteins, according to Drugbank. Panel **B**: Functional analysis of proteins identified as agalsidase α (upper pie), agalsidase β (middle pie), and endogenous AGAL (lower pie)-interacting proteins clusterized according to biological processes and cell components to which they belong.

In the main experiment, IF cells were treated with 3 µg/mL rh-AGAL for 6 h, then collected and lysed. Cell extracts underwent co-immunoprecipitation, and the precipitates were analyzed via mass spectrometry to identify the interactors. The experiment identified 99 and 112 putative interacting proteins in IF cells treated with agalsidase α or agalsidase β, respectively (Figure 2A and Supplementary File S1). These interactors were clusterized according to their biological functions as reported in the literature and the UniProt database. Results were represented in pie graphs (Figure 2B).

Similar patterns of AGAL interactors were recorded in the cells treated with different rh-AGALs. Interestingly, the classification based on the biological function showed that the class of proteins involved in endocytosis and trafficking was quite populated. This enrichment is somehow expected, considering that the therapeutic approach relies upon the internalization of the recombinant enzyme administered.

Both rh-AGALs interact with many proteins involved in clathrin- or caveolin-dependent endocytic processes. Among them, it is worth mentioning AP2A1 and AP2B1 (subunits of the adaptor complex [40]), and SNX18 (member of sorting nexins [41]) as participants in the endocytosis clathrin-dependent internalization process.

CAVIN1 [42], its interactor dynamin-related protein EHD2 [43], CAV1 [44], RHOG [45] and ARHGDIA [46] are involved in the formation and remodeling of caveolae. EHD2 and CAVIN1 are interactors of both rh-AGALs, while CAV1 and ARHGDIA have been found among agalsidase β interactors. This result might suggest a significant contribution of this pathway to the agalsidase β internalization process if compared to agalsidase α. 

Interestingly, ACTB and PRDX6, which we found among agalsidase β interactors, had previously been described as being reduced in the plasma of FD patients upon enzyme replacement therapy with agalsidase β [47].

Galectin-1 (*LGALS*), a common interactor, had been identified as an up-regulated protein in FD [48,49]. Galectin-1 is an inflammatory marker, a protein with many different biological activities, and one of the master regulators of the immune response [50].

Among protein categories involved in vesicular transport, we also found MYO1B and MYO1C (agalsidase α) and MYO1D (agalsidase α and agalsidase β). These entries belong to unconventional myosins, a class of single-headed myosin motors that participate in exocytosis, endocytosis, and trans-Golgi network trafficking by tethering vesicles to the cortical actin filaments [51].

Interestingly, MYO1C [52] and MYO1B [53] are involved in the autophagic pathway. Defective autophagic flux has been associated with several lysosomal storage diseases (LSDs), including Fabry disease [54]. In particular, MYO1C downregulation has been linked to blocking autophagosome–lysosome fusion [55].

The differences in the interaction profiles of agalsidase β and agalsidase α, especially those concerning the intracellular trafficking of rh-AGAL, might shed light on the different posology needs for the two approved rh-AGALs [22].

In this sense, different proteins associated with the exocytic pathway were identified as rh-AGAL interactors: MYO6, RAB8A, OPTN [56]. However, it should be emphasized that all these markers are present together, exclusively within the agalsidase β interactome, while only MYO1C is in common with agalsidase α. This different enrichment in the interactions suggests the possibility of an increased elimination of agalsidase β by activating the exocytic pathway. This phenomenon could explain the need for a higher dosage of agalsidase β compared to agalsidase α.

Finally, we analyzed the interactome of the endogenously produced AGAL. Finding interactors of internalized, exogenously added rh-AGALs can benefit the patient who does not express the enzyme at all and necessitates ERT, whereas finding interactors of endogenous AGAL can benefit those patients who express a mutated AGAL whose deficient activity can be potentiated by PCT.

We started from the same cell line to minimize the background effect and appropriately compare data with those obtained with the rh-AGALs. IF cells were thus stably transfected with endogenous *GLA* (IF-GLA), as in [32], and then analyzed as described above. For endogenous AGAL, only 48 putative interactors were identified; in this case, 17% of them belonged to the endocytosis and trafficking typology (Figure 2A and Supplementary File S1). AGAL is synthesized in the ER, transported to the Golgi apparatus and tagged with mannose-6-phosphate for delivery to the lysosomes. Thus, the abundance of this class of interactors was expected.

The classification based on the biological function showed that proteins involved in folding were relatively abundant for endogenous and rh-AGALs. This evidence is in line with recent findings on the use of proteostasis regulators to overcome the protein processing defects occurring in FD [57] and, more generally, in lysosomal storage disorders [58].

BiP (*HSPA5*), an endoplasmic reticulum chaperone, was among the protein folding-related interactors. Recently, we described the effect of acetylsalicylic acid as an enhancer of PCT for FD, hypothesizing that the interaction with BiP is part of the mechanism of action of ASA in FD [32]. We also demonstrated the beneficial effect of curcumin for various AGAL mutants under mono- and combined therapies with pharmacological chaperones [59]. Interestingly, BiP is also described as sensitive to curcumin [60]. The regulation of cytoskeleton proteins found among the AGAL interactors, such as TUBB and VIM, was previously associated with curcumin treatment, too [61,62,63,64]. Furthermore, myosin light polypeptide 6 (*MYL6*), which is involved in the cytoskeleton and related to the extracellular exosome and membrane trafficking, was previously identified as differentially expressed in FD [65]. Looking at the network intersection of each rh-GAL and endogenous protein separately (agalsidase α versus endogenous, and agalsidase β versus endogenous), we can note that agalsidase α shares a specific group of interactors, contrarily to agalsidase β. This group of proteins is mainly reinforced in the cytoskeleton and transport machinery including among VIM, ACTN1 and MYO1C. Indeed, VIM has been linked to the trafficking processes at the level of the late endosomes [66,67]. The presence of MYO1C in this subgroup is also interesting. As reported above, this protein has a role in autophagosome–lysosome fusion, and the combination of ERT and autophagy inducers has been demonstrated to improve the therapeutic outcomes in Pompe disease [68].

The described results mutually support our previous papers and strengthen the importance of drug repositioning in rare diseases. Drug repositioning is the use of an approved drug for new therapeutic purposes [69]. It helps reduce the so-called “time between bench and bedside” and the research-related costs, strongly impacting the risk of failure, which jumps from 95% with de novo drug discovery to 45% with drug repositioning [70,71,72].

Aiming to evaluate and prime repositioning strategies, we mined our interactomes for entries known to interact with approved drugs (Figure 2A, red nodes) and built protein/drug association tables (Supplementary File S2). Found drugs may act as ERT modulators, giving the list potential to pave the way to new combined therapies for FD. Furthermore, interactors of endogenous AGAL might represent therapeutic targets in the case of FD missense mutations not requiring ERT. For this reason, the list of druggable endogenous AGAL interactors reported in Supplementary Table S2 doubles as a starting point to improve the screening of approved drugs for repositioning.

Interestingly, about 60% of the druggable proteins were directly or indirectly linked to autophagy in the literature (manual check on PubMed performed on 29 December 2022). We found this association of the utmost importance since autophagy is described as one of the main pathogenic mechanisms of FD, together with lysosomal dysfunction and altered lipid metabolism [73]. Last but not least, during the last two decades, autophagy has been a very popular topic that has spawned a large body of scientific literature [74,75,76,77].

On the one hand, excessive autophagy can increase metabolic stress and cell death; on the other, a balanced level of autophagy ensures the degradation of denatured proteins and nucleic acids in damaged, ageing cells and organelles. In doing so, it provides raw materials for cell regeneration and repair [77]. In a therapeutic context, this duality translates into the need to thoroughly study mechanisms associated with autophagy and carefully identify inhibitors/activators of this process [73,78,79].

## 3. Materials and Methods

### 3.1. Cell Cultures

Immortalized patient-derived fibroblasts carrying a large deletion in *GLA* exons 3 and 4 (IF) were obtained from the Telethon Biobank and eventually stably transfected as previously described [32]. Cells were cultured in RPMI 1640 medium, supplemented with 10% FBS, 2 mM glutamine, 0.5 mg/mL penicillin, 0.5 mg/mL streptomycin, and non-essential amino acids at 37 °C in 5% humidified CO_2_. Treatments were performed as described in the captions.

### 3.2. Enzymatic Activity Assays

Fibroblasts from 90% confluent six-well plates were collected in Roche M cOmplete lysis buffer (Merck) and centrifuged for 10 min at 14,000× *g*. AGAL enzymatic activity assay was performed as described in [80] with the modifications described in Monticelli et al. [32].

Agalsidase α (Replagal^®^, Shire Human Genetic Therapies, Inc., Chineham, UK) was diluted in pre-warmed FBS (not previously heat-inactivated) in the presence of 0–10 mM galactose, and incubated at 37 °C. Aliquots were withdrawn at 20, 40 and 60 min and AGAL activity was measured using the synthetic substrate paranitrophenyl-α-galactopyranoside (Merck) 14 mM in potassium acetate buffer at pH 5.2 and 37 °C. The enzymatic assay was performed discontinuously, sampling and stopping the reaction at 0, 3, 6, 9 and 12 min, by addition of 0.5 M Na_2_CO_3_. The absorbance of para-nitrophenolate at 405 nm was then measured.

### 3.3. Purification, Identification, and Functional Analysis of AGAL Interactomes

Immortalized patient-derived fibroblasts (IF) incubated for 6 h with 3 µg/mL agalsidase α (Replagal^®^, Shire Human Genetic Therapies, Inc., England) or agalsidase α (Fabrazyme^®^, Genzyme Corp., Cambridge, MA, USA), and IF cells stably transfected with wt-*GLA* (IF-GLA, [32]) were lysed in 50 mM Tris HCl at pH 6.5, 150 mM NaCl, 0.1% Triton X-100, 2.5 mM KCl and a cocktail of protease inhibitors (Roche). Protein extracts were quantified by Bradford assays (Biorad), pre-cleared by incubation with Dynabeads Protein-G (Thermo), and immunoprecipitated using a specific rabbit polyclonal antibody anti-hAGAL. Proteins were eluted in Glycine pH 2.5 and fractionated by SDS-PAGE. The whole lanes were cut in 96 bands and in situ digested with trypsin, according to [81]. The obtained mixtures were analyzed by LC-MS/MS, using a LTQ Orbitrap XL system (Thermo Fisher) equipped with a nano-Easy II HPLC [82]. Peptide analysis was performed using the data-dependent acquisition (DDA) of one MS scan (mass range from 400 to 1800 m/z) followed by MS/MS scans of the five most abundant ions in each MS scan. The mass spectrometry proteomics data have been deposited in the ProteomeXchange Consortium via the PRIDE partner repository with the dataset identifier PXD039168 [83].

Protein identifications were carried out using the MASCOT software (Matrix Science, Boston, MA, USA) by setting the parameters as reported in [84]. All identified proteins were clustered according to their biological functions as reported in the literature and UniProt schedules.

### 3.4. Miscellaneous

Protein concentration was determined using the Bradford method and BSA as the standard [85]. Immunoblots were performed under standard conditions, as described in [86].

Starting from the list produced via the interactome analysis, we extracted the Uniprot ID and used the Uniprot ID mapping functionality (https://www.uniprot.org/id-mapping, accessed on 2 May 2022) to access UniProtKB entries. We then downloaded each entry’s drug association (as DrugBank ID, DBid). Finally, we converted the DBid into the drug name using the drugbank vocabulary from https://go.drugbank.com/releases/latest#open-data (accessed on 2 May 2022). The entire process was scripted in R [87] using the tidyverse family of packages [88] and is available as Supplementary File S3.

## 4. Conclusions

This brief report explores two approaches that could be developed to improve ERT based on its combination with small molecules acting at different cellular levels. Combined therapies recently arose for rare diseases [32,89,90,91,92], and in particular, the US Food and Drug Administration (FDA) approved one of them for treating cystic fibrosis [93]. Thus, they represent a very promising approach.

Our preliminary data, on the one hand, hints that galactose, a low-affinity pharmacological chaperone (PC), can prolong the stability of the internalized rh-AGAL. PCs act by explicitly binding a target protein and stabilizing it. In the case of mutant proteins, the stabilization prevents unfolding-derived degradation; likewise, in the case of wt proteins, as it is for the administration of rh-AGAL, the increased stability would prolong the half-life. We demonstrated that in an FD cell model, the administration of galactose lengthens the presence of internalized rh-AGAL over seven days. It is worth noticing that besides the intracellular stabilization of rh-AGAL by galactose, a stabilizing and/or activating effect(s) in plasma would contribute to the benefits provided by galactose. This kind of stabilization has been described for DGJ, requiring in vivo experiments to be assessed. Thus, moving from cell in vivo models will be necessary, following what is described for DGJ [26,94]. Mice models would allow us to perform an accurate study committed to analyzing this aspect and optimizing dosages and timing, and could evaluate the applicability and definitively demonstrate the usefulness of the rh-AGAL/galactose combination in treating FD. Moreover, such a model would also allow us to compare galactose and DGJ effects in vivo, based on substrate clearance efficiency. In fact, as previously discussed, the different Ki for DGJ and galactose make DGJ a stronger stabilizer but also a stronger inhibitor [28]. We strongly believe galactose may optimize the balance between these classical pharmacological chaperones’ stabilizing and inhibiting activity.

On the other hand, we analyzed the interactome of human endogenous and rh-AGAL and produced a list of druggable proteins among the AGAL partners. This list represents a starting point for future screening. Selected FDA-approved drugs in our list could guide the choice of ERT-potentiating partners. It is also interesting to speculate that interactors might represent reducers rather than adjuvants. In fact, besides the stability extension, AGAL could undergo a faster degradation or secretion via a drug’s effect on its interactors. These aspects should be carefully evaluated in pre-clinical research, and investigated in clinical trials to optimize FD therapies and address the unmet needs of FD patients.

Finally, the interactors identified for the endogenous AGAL can represent therapeutic targets in FD caused by missense mutations.

## Data Availability

The data presented in this study are available in the article and supplementary material. Proteomics data are available as PXD039168 on ProteomeXchange.

## Supplementary Materials

The following supporting information can be downloaded at: https://www.mdpi.com/article/10.3390/ijms24054548/s1.

## Abbreviations

AGAL	α-galactosidase
CHO	Chinese hamster ovary cells
DGJ	1-deoxygalactonojirimycin
ERT	Enzyme Replacement Therapy
FBS	Fetal Bovine Serum
FD	Fabry Disease
FDA	Food and Drug Administration
Gb3	Globotriaosylceramide
IF	FD patient-derived immortalized fibroblasts
IF-NULL	FD patient-derived immortalized fibroblasts stably transfected with the empty vector
IF-GLA	FD patient-derived immortalized fibroblasts stably transfected with a plasmid encoding wt-*GLA*
PC	Pharmacological Chaperone
PCT	Pharmacological Chaperone Therapy
rh-AGAL	Recombinant human—α-galactosidase
