# Supplementary figures and images for: Enzyme Replacement Therapy for FABRY Disease: Possible Strategies to Improve Its Efficacy
Source: Int J Mol Sci. 2023 Feb 25;24(5):4548. doi: 10.3390/ijms24054548 (PMC10003632; doi:10.3390/ijms24054548)

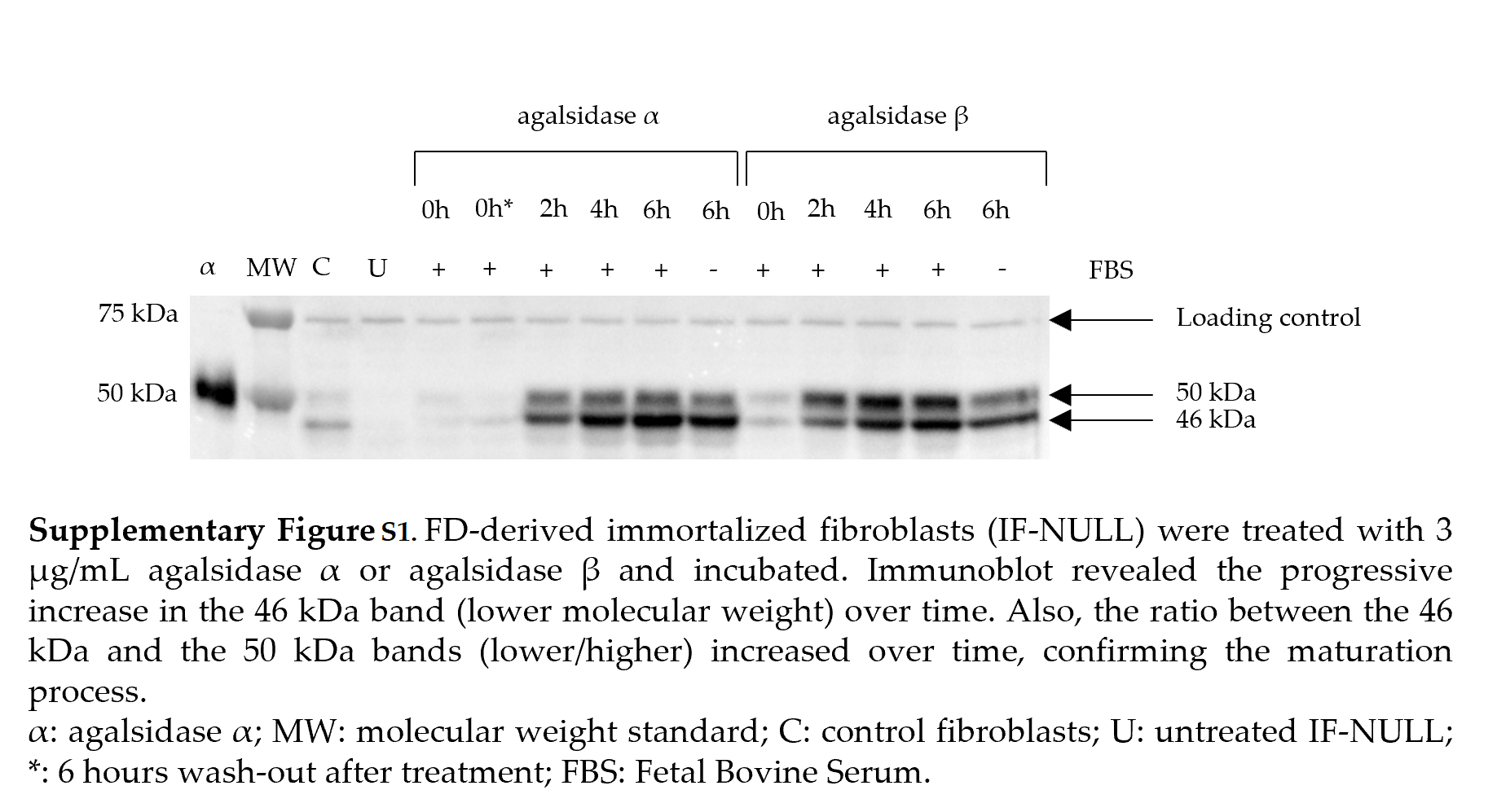

Supplement: Supplementary file 1 [file ijms-24-04548-s001.zip › Supplementary Figure S1.png]

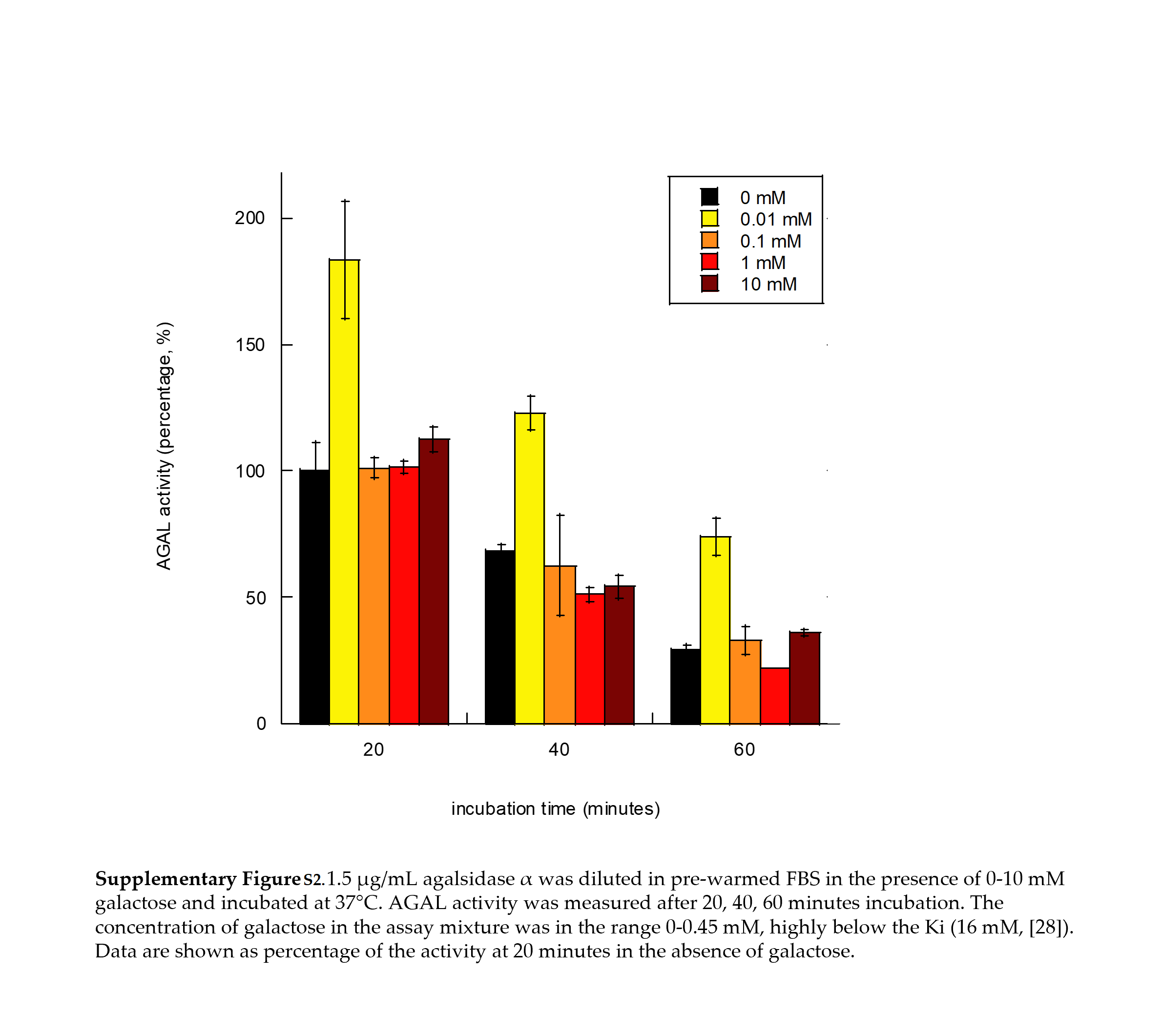

Supplement: Supplementary file 1 [file ijms-24-04548-s001.zip › Supplementary Figure S2.png]
